# Supplementary material for: Defective splicing of Y-chromosome-linked gigantic genes contributes to hybrid male sterility in Drosophila
Source: Mol Biol Evol. 2026 Feb 16;43(3):msag045. doi: 10.1093/molbev/msag045 (PMC12965833; doi:10.1093/molbev/msag045)
Supplement: msag045_Supplementary_Data [file msag045_supplementary_data.zip › Supplementary Figures - hybrid Yloop.pdf]

Supplementary Figures and Table

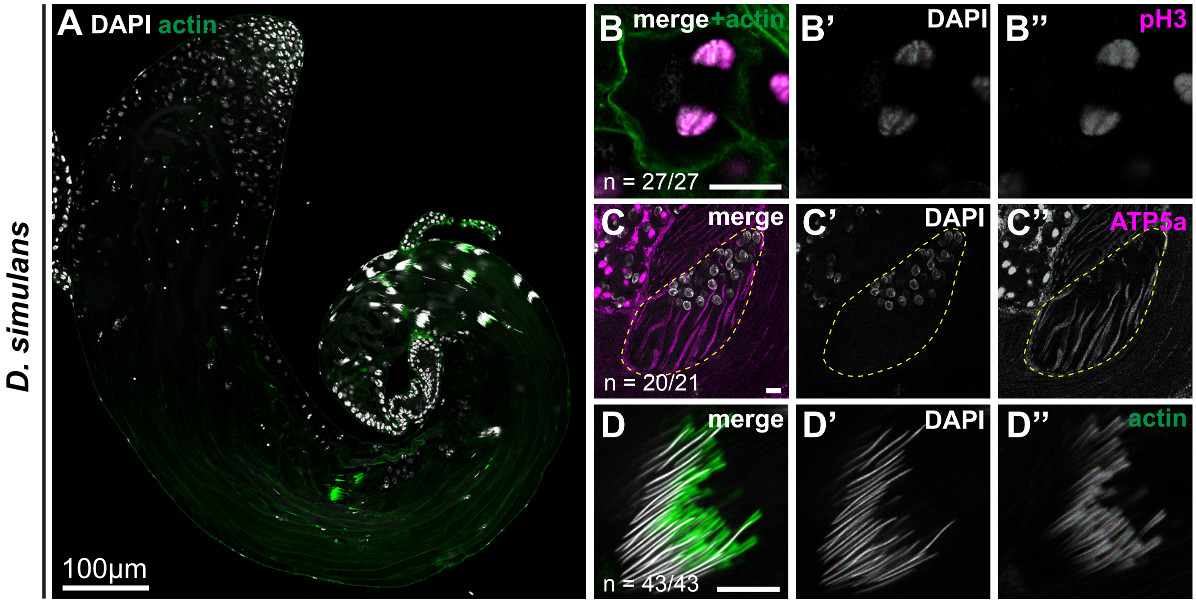

Supplementary Figure S1: *D. simulans* spermatogenesis

A-D. *D. simulans* testis and germ cells. Low magnification view of the whole testis (A) stained for F-actin (green, stained with phalloidin-Alexa 488) and DAPI (gray). Meiotic anaphase (B) stained for phospho-H3 ser10 (magenta) and DAPI (gray). Early elongating spermatids (C) stained for ATP5a (magenta, mitochondria) and DAPI (gray). Needle stage spermatids (D) stained for F-actin (green, stained with phalloidin-Alexa 488, individualization complex). Scale bar: 10µm, unless otherwise noted.

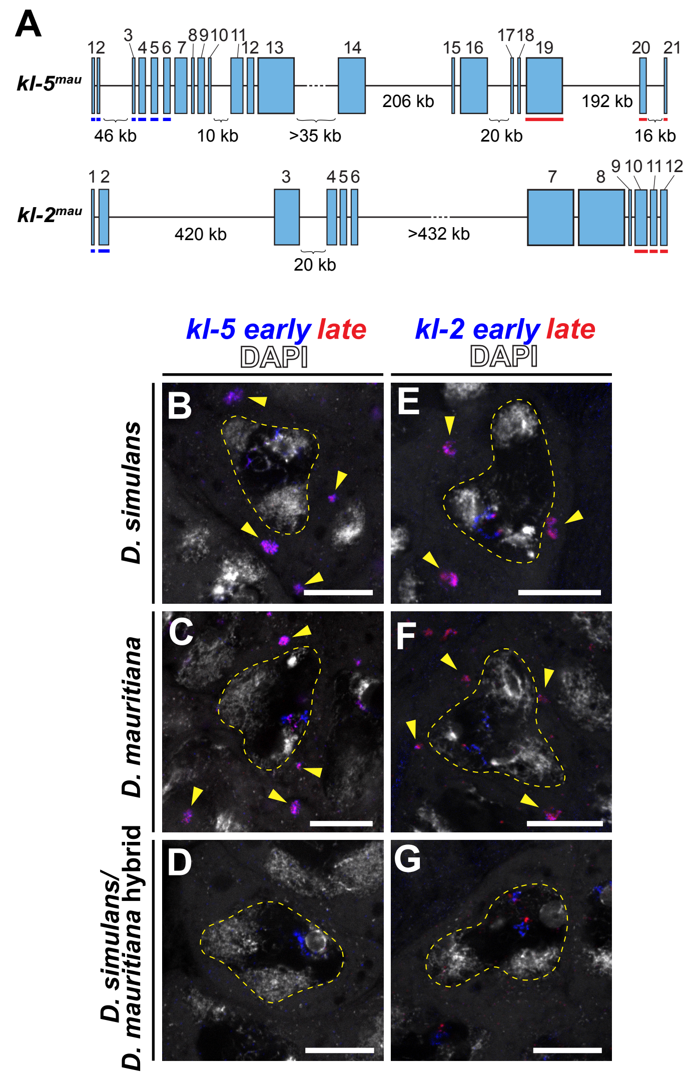

Supplementary Figure S2: Expression of *kl-2* and *kl-5* in the spermatocytes of the hybrids and parental species.

A. *D. mauritiana kl-5* (*kl-5*^{mau}) and *kl-2* (*kl-2*^{mau}) gene structure. The positions of smRNA FISH probes are indicated.

B-F. RNA in situ hybridization for *kl-5* transcripts in late-stage spermatocyte nuclei of *D. simulans* (B), *D. mauritiana* (C) and *D. simulans/D. mauritiana* hybrids (D). Spermatocyte nuclei are indicated by dashed lines. Arrowheads indicate cytoplasmic kl-granule containing mature *kl-5* mRNA. Blue: *kl-5* early exons. Red: *kl-5* late exons. Gray: DAPI. Scale bar: 10µm.

E-G. RNA in situ hybridization for *kl-2* transcripts in late-stage spermatocyte nuclei of *D. simulans* (E), *D. mauritiana* (F) and *D. simulans/D. mauritiana* hybrids (G). Spermatocyte nuclei are indicated by dashed lines. Arrowheads indicate cytoplasmic kl-granule containing mature *kl-2* mRNA. Blue: *kl-2* early exons. Red: *kl-2* late exons. Gray: DAPI. Scale bar: 10µm.

A

RT-PCR Target	Amplicon size (bp)
kl-3 exon 5-exon 6	512
kl-3 exon 5-exon 4	506
kl-3 exon 5-exon 2	410
kl-5 exon 19-exon 20	554
kl-5 exon 19-exon 17	504
ory exon 7-exon 8	398
ory exon 7-exon 5	301
ccy exon 4-exon 5	422
ccy exon 4-exon 3	600

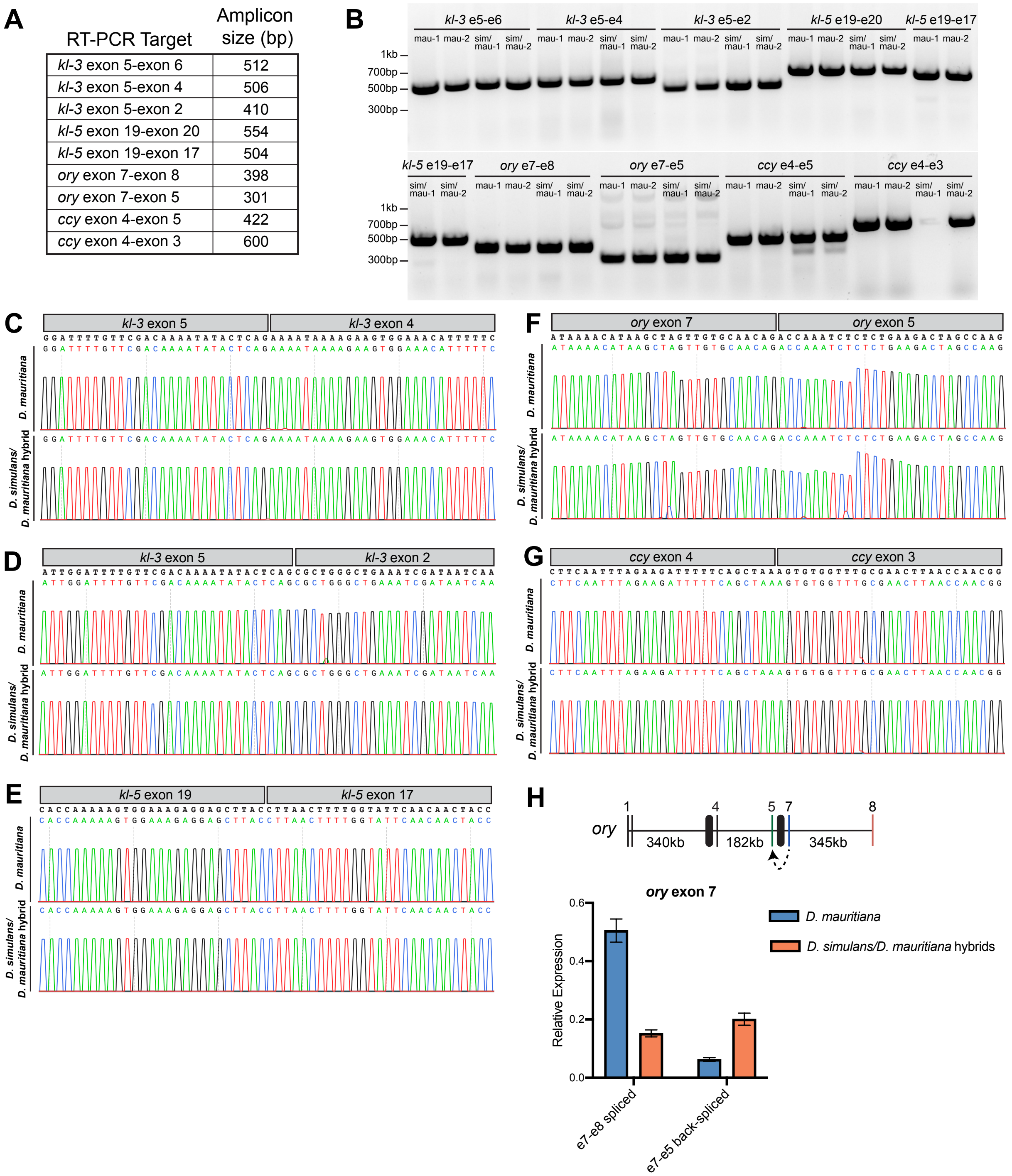

Supplementary Figure S3: Validation of back-splicing products.

A-B. RT-PCR for back-spliced transcripts. (A) List of expected PCR amplicon sizes. Primer sequences can be found in Supplementary_file_3. (B) RT-PCR products for two biological replicates of each *D. mauritiana* and *D. simulans/D. mauritiana* hybrids. *CCY* e4-e3 failed to amplify in one of the hybrid replicates.

C-G. Oxford Nanopore sequencing of the PCR products generated in (A-B) aligned to the corresponding exon-exon junction. Only the sequence immediately around the junction is shown.

H. RT-qPCR results of spliced vs. back-spliced products involving *ORY* exon 7. Schematic of the exon-intron structure surrounding *ory* exon 7 is shown in the upper panel. Exons are represented by vertical lines and labeled by exon number. Introns are represented by horizontal lines; a dotted line indicates a gap in the genome assembly. RT-qPCR results were normalized to GAPDH. Error bars represent SEM across three technical replicates. (See Supplementary_file_3 for raw CT values and biological replicates.)

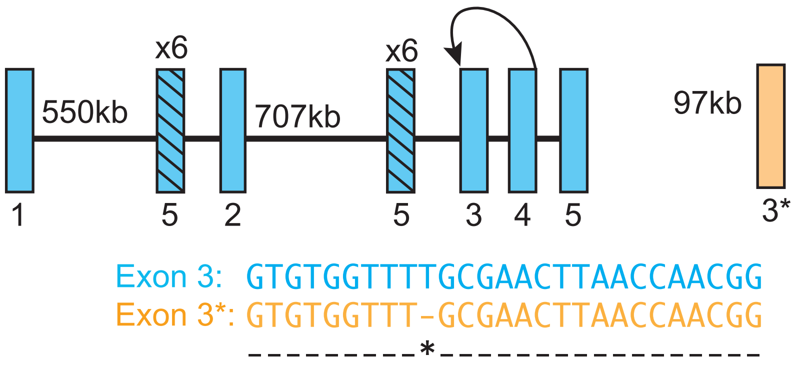

Supplementary Figure S4: Structure of CCY and its back-splicing patterns
CCY gene contains multiple duplicated exons. Exon 4 - exon 3 spliced product was detected, and this may be explained by exon 4 - exon 3 back-splicing or exon 4- exon 3* forward-splicing (exon 3* is located 97 kb downstream of CCY's terminal stop codon). However, based on SNP (shown at the bottom), it was determined that exon 4 – exon 3 back-splicing

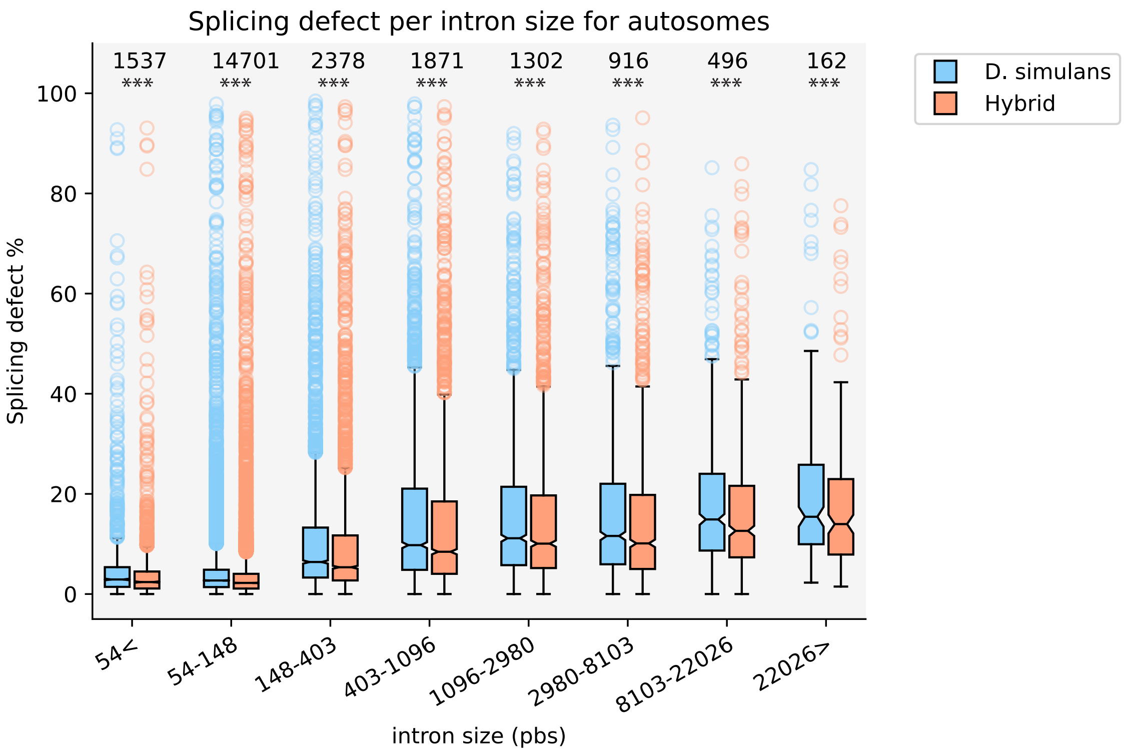

Supplementary Figure S5: Frequency of splicing defects for autosomal genes.
Frequency of splicing defects (100% - correctly spliced reads/all reads) for autosomal genes based on the size of introns. Introns were binned by their size (logarithmic scale with the base of e). Stars indicate statistical significance between *D. simulans/D. mauritiana* hybrids and *D. mauritiana* (Supplementary_file_2 for statistics details.)

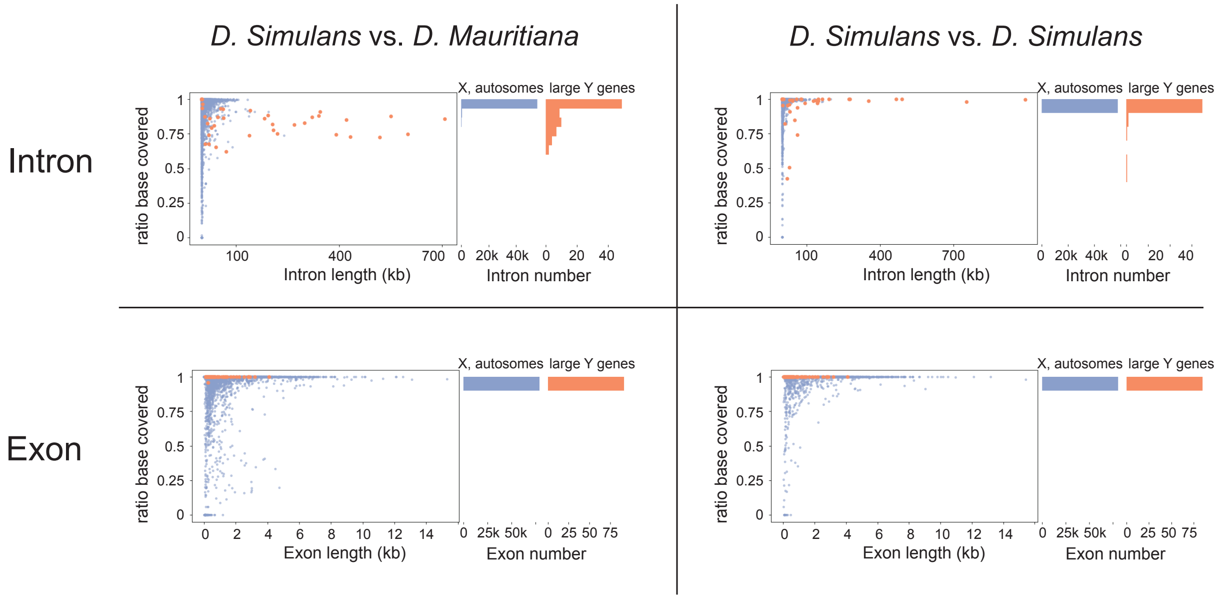

Supplementary Figure S6: estimation of DNA sequence conservation between *D. simulans* and *D. mauritiana* for exons and introns

Ratio of covered bases for genomic features (intron: top row, exon: bottom row) by short read DNA sequencing from *D. simulans* aligned against either *D. mauritiana* genome (left) or *D. simulans* genome (right). Features from X and autosomes are shown in blue, and large Y genes in orange. Histograms (right to each scatter plot) indicate the distribution of introns based on the conservation (Y axis).

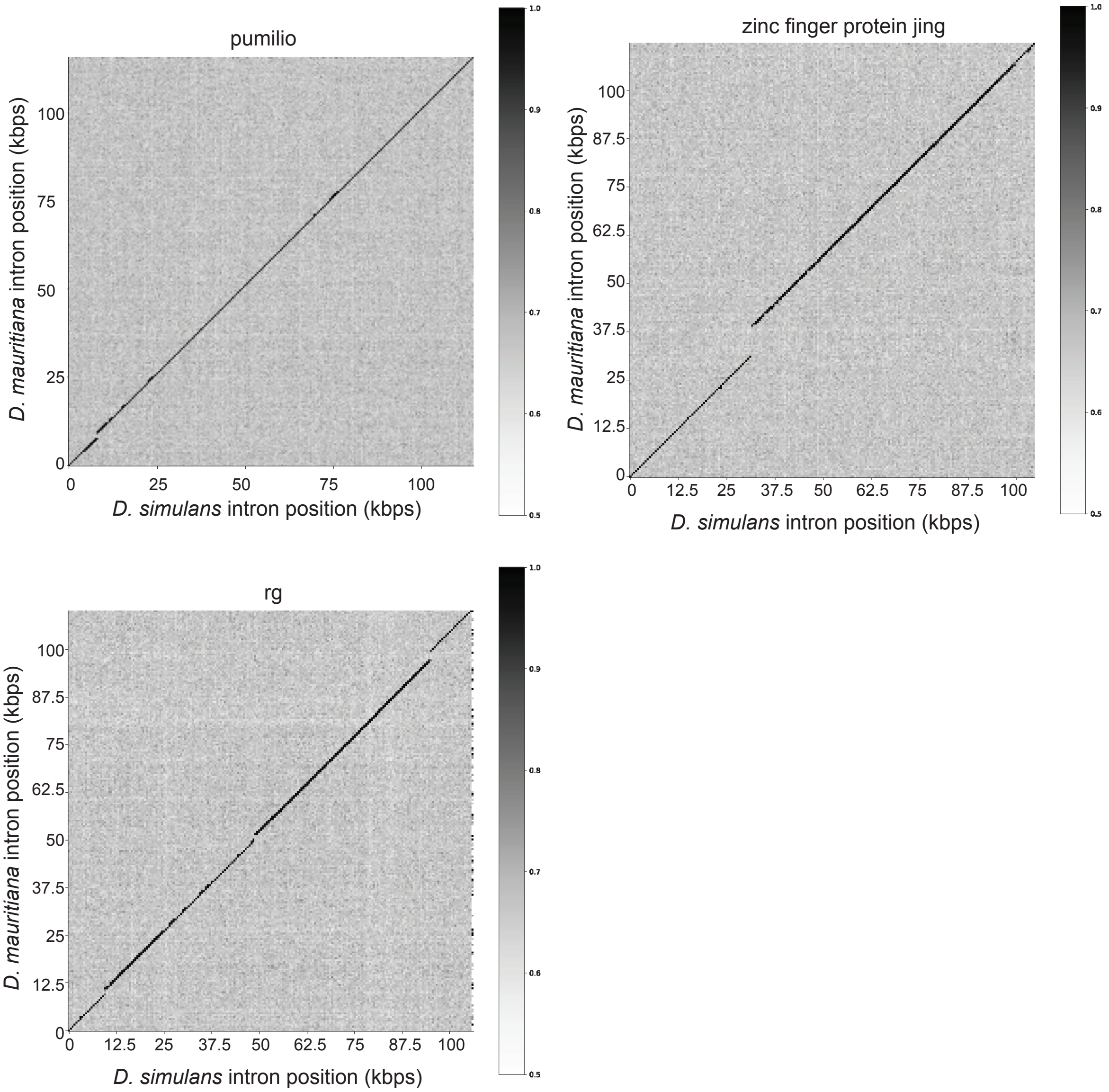

Supplementary Figure S7: Large introns of autosomal genes are conserved.
There are only three autosomal genes that have large introns (>100kbps) and are expressed in the testes. They are well-conserved. Window size is 500 bps, and alignment was performed with the Needleman-Wunsch algorithm. Scoring: ECDNA matrix, gap open -10 and gal extension -0.5.

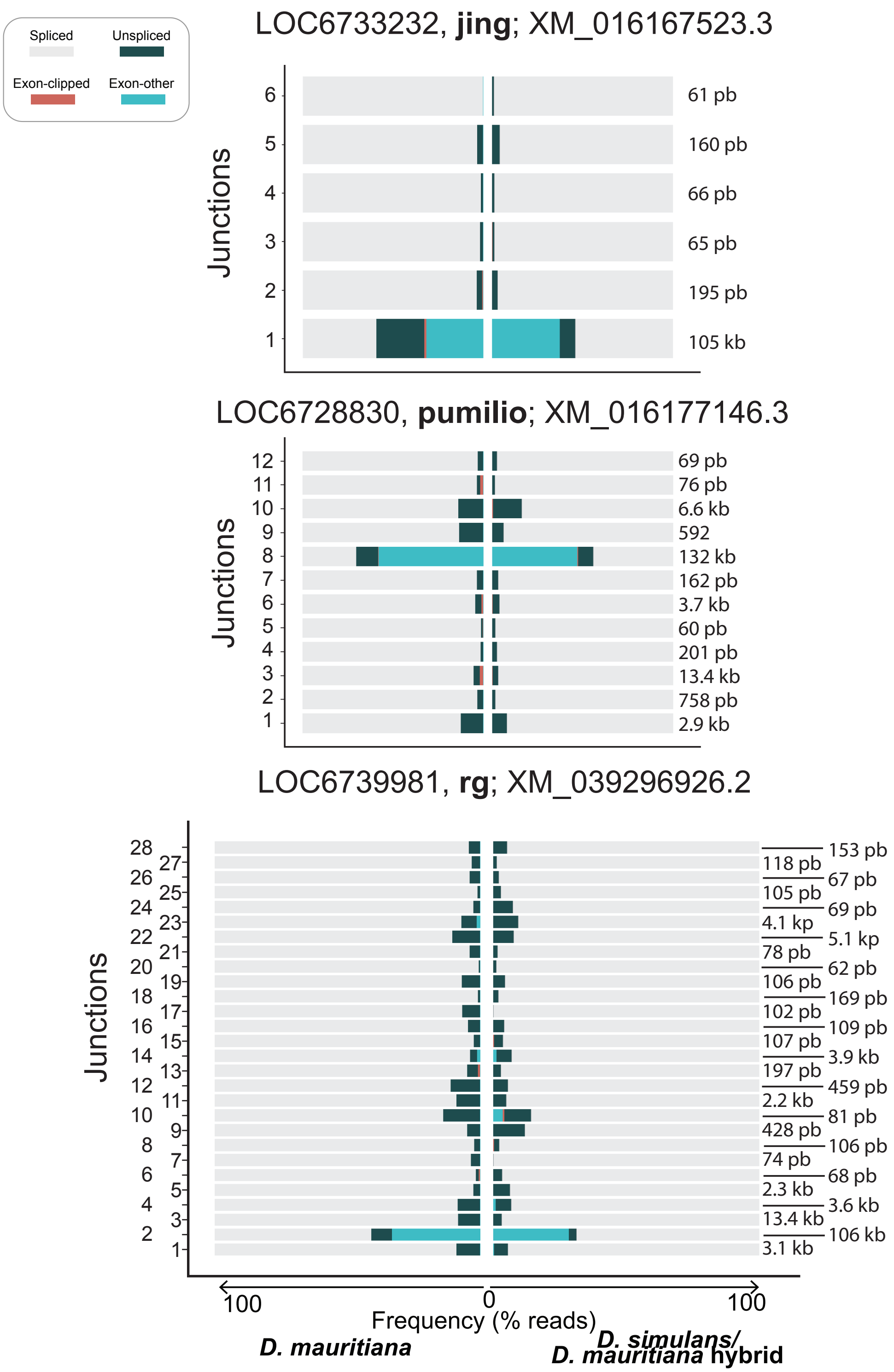

Supplementary Figure S8: Sequence read analysis for each exon end of the three genes with large introns linked to the X chromosome or autosomes. Large introns of these genes did not exhibit a statistically significant difference between *D. mauritiana* and *D. simulans/D. mauritiana* hybrids.

Blanks

	Dmel	Dsim	Dmau	Dsec
Dmel	-	54.87	54.97	57.34
Dsim	-	-	91.94	54.73
Dmau	-	-	-	53.39
Dsec	-	-	-	-

Heph

	Dmel	Dsim	Dmau	Dsec
Dmel	-	66.52	73.39	94.44
Dsim	-	-	89	67.18
Dmau	-	-	-	74
Dsec	-	-	-	-

Maca

	Dmel	Dsim	Dmau	Dsec
Dmel	-	78.09	79.68	78.88
Dsim	-	-	94.72	95.08
Dmau	-	-	-	96.34
Dsec	-	-	-	-

Supplementary Table S1: Protein sequence divergence of Blanks, Heph and Maca
% identity from amino acid alignment using blosum62 and gap open -10 and gap extension 0.5
