# Supplementary material for: Defective splicing of Y-chromosome-linked gigantic genes contributes to hybrid male sterility in Drosophila
Source: Mol Biol Evol. 2026 Feb 16;43(3):msag045. doi: 10.1093/molbev/msag045 (PMC12965833; doi:10.1093/molbev/msag045)
Supplement: msag045_Supplementary_Data [file msag045_supplementary_data.zip › Supplementary_file_3.pdf]

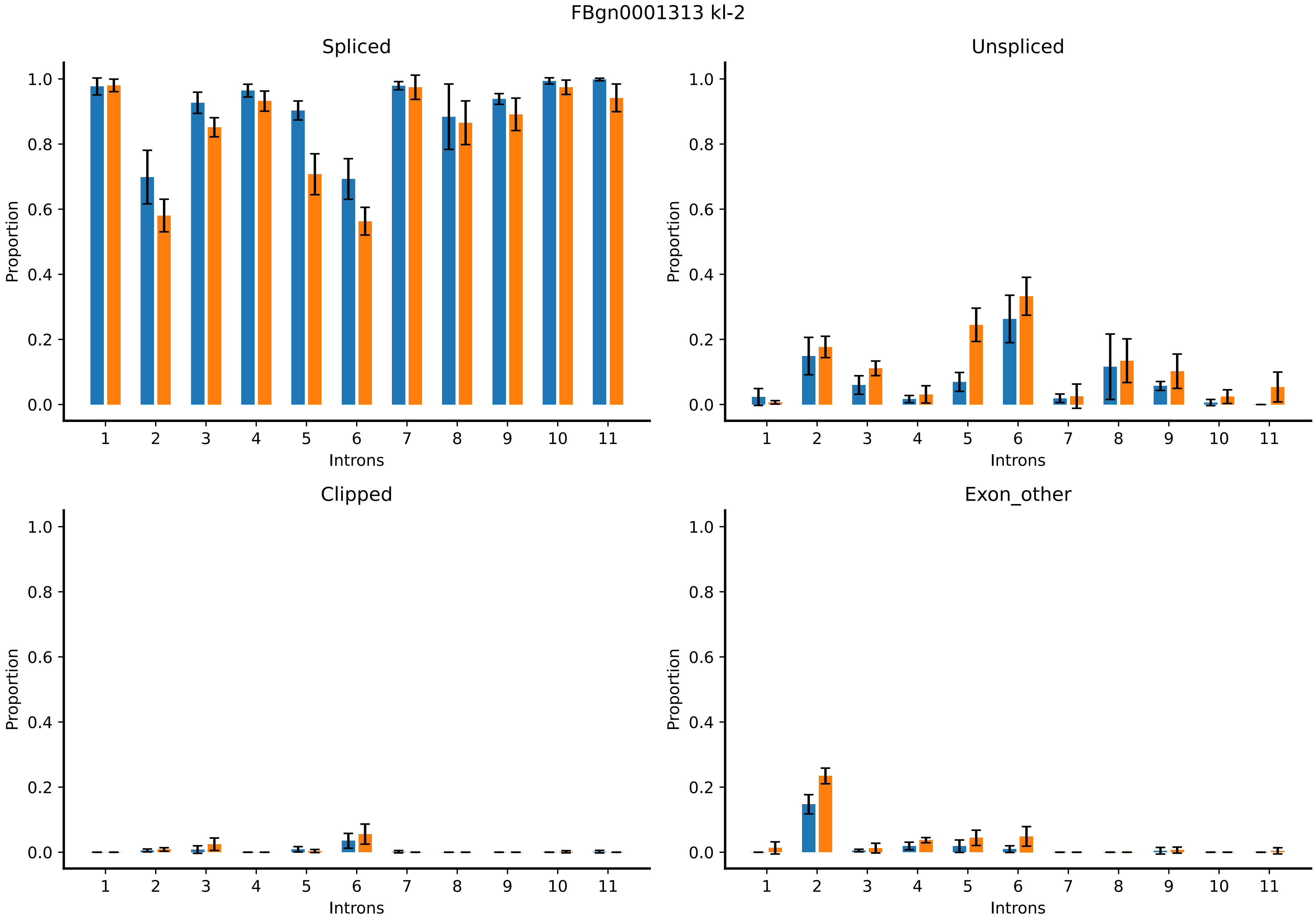
FBgn0001313 kl-2
Spliced
Unspliced
Clipped
Exon_other
Proportion
Introns
1.0
0.8
0.6
0.4
0.2
0.0
1
2
3
4
5
6
7
8
9
10
11

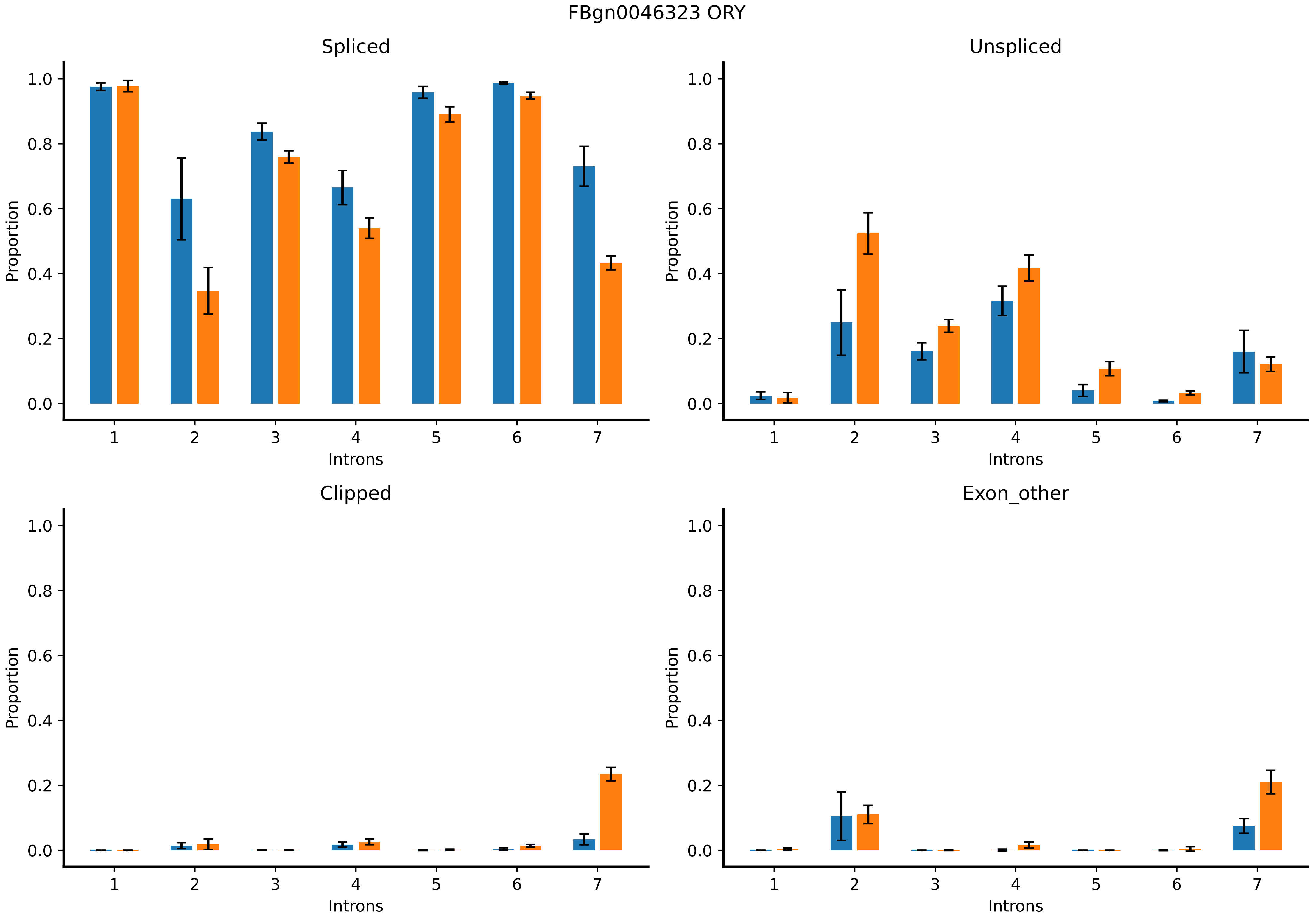
FBgn0046323 ORY
Spliced
Unspliced
Clipped
Exon_other
Proportion
Introns
0.0
0.2
0.4
0.6
0.8
1.0
1
2
3
4
5
6
7

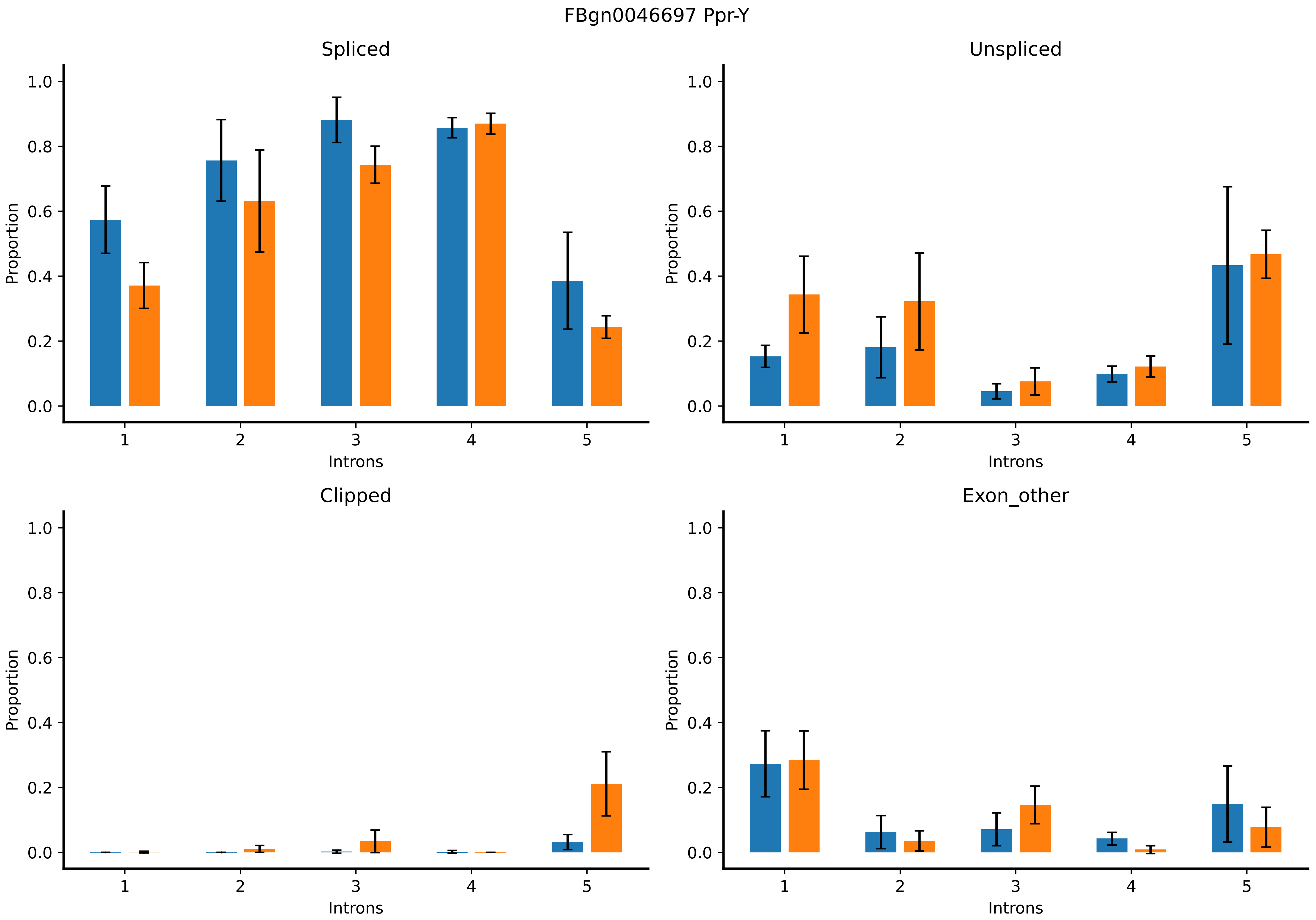
FBgn0046697 Ppr-Y
Spliced
Unspliced
Clipped
Exon_other
Proportion
Introns
1.0
0.8
0.6
0.4
0.2
0.0
1
2
3
4
5

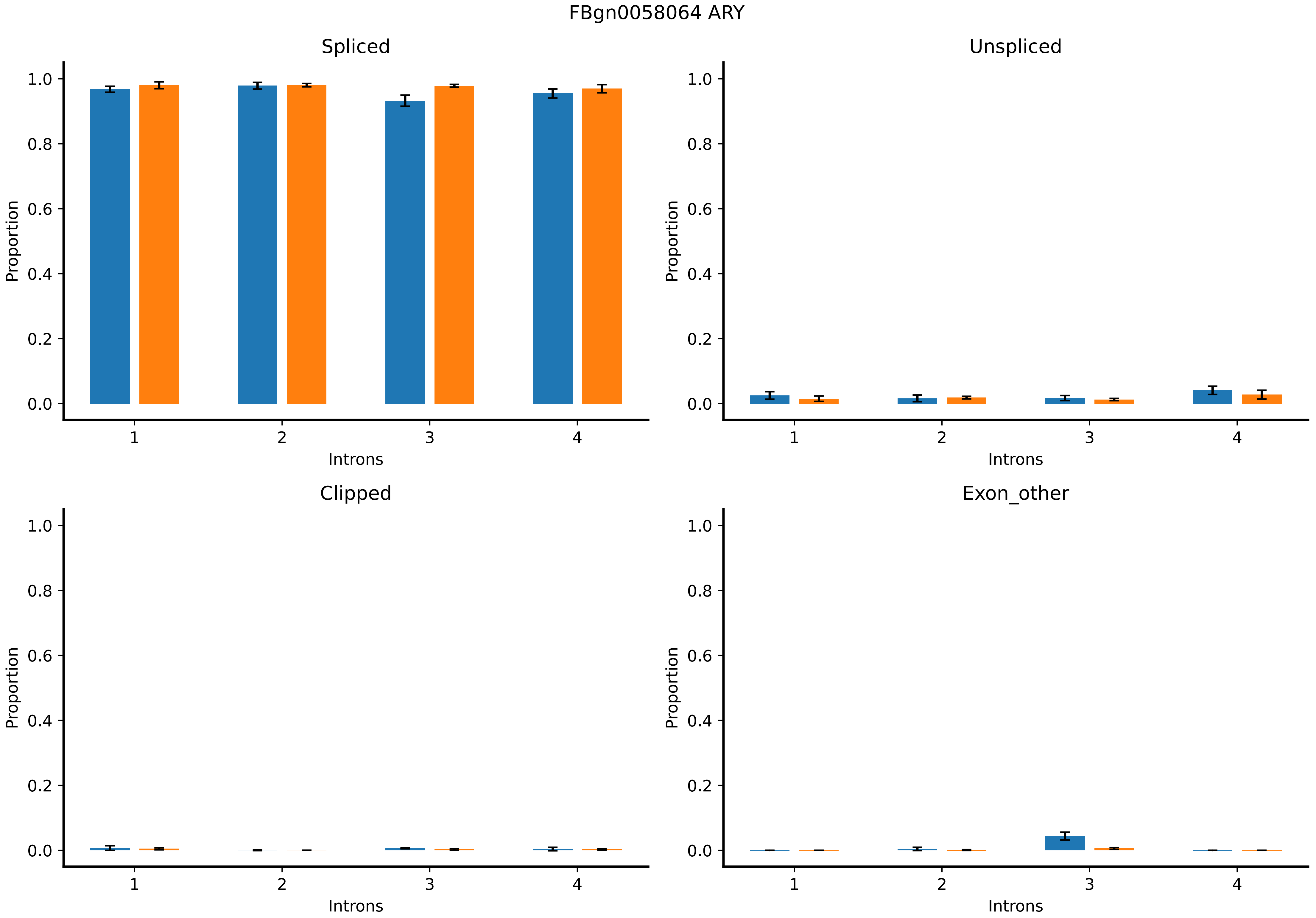
FBgn0058064 ARY
Spliced
Unspliced
Clipped
Exon_other
Proportion
Introns
0.0
0.2
0.4
0.6
0.8
1.0
1
2
3
4

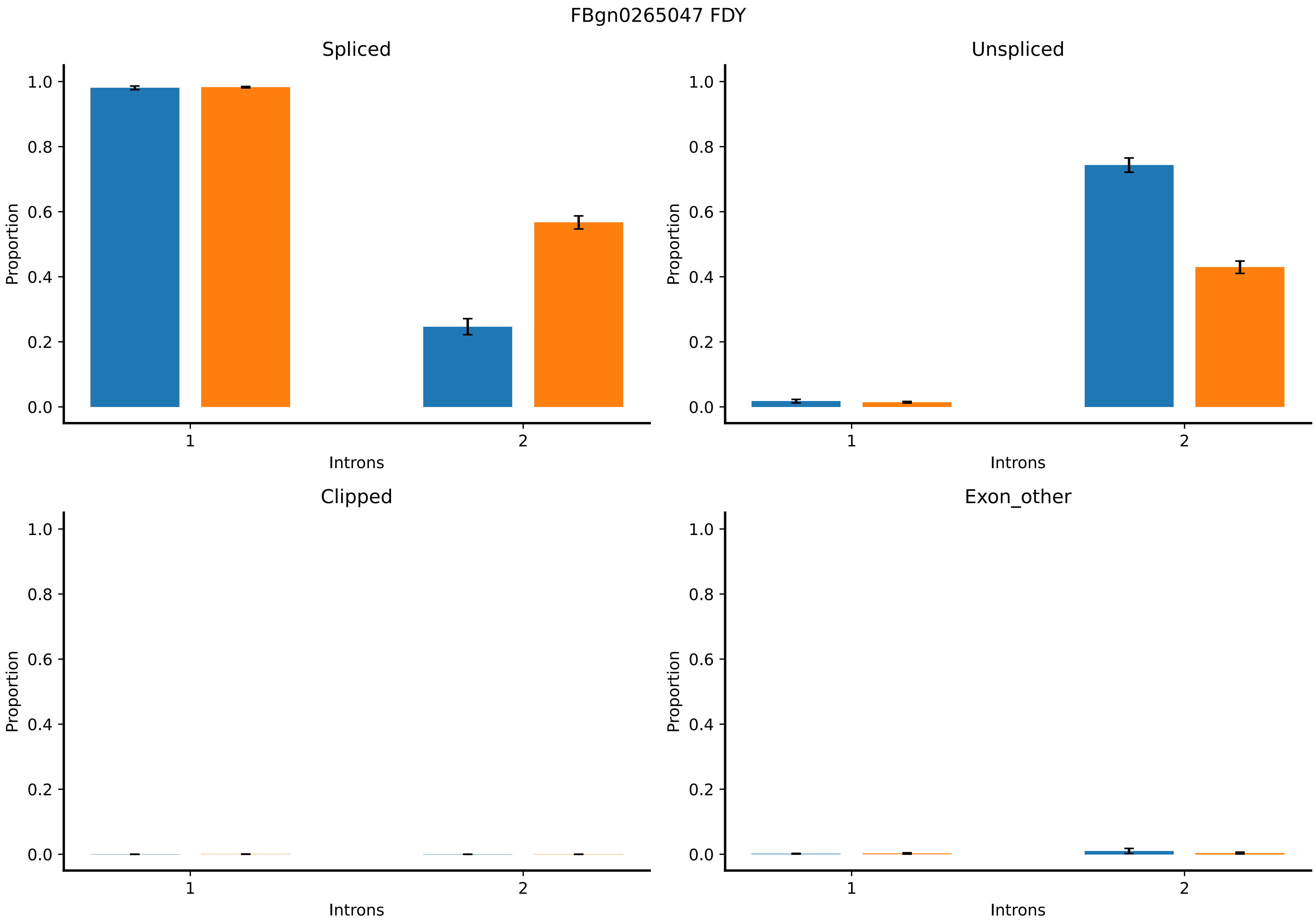
FBgn0265047 FDY
Spliced
Unspliced
Clipped
Exon_other
Proportion
Introns
1.0
0.8
0.6
0.4
0.2
0.0
1
2

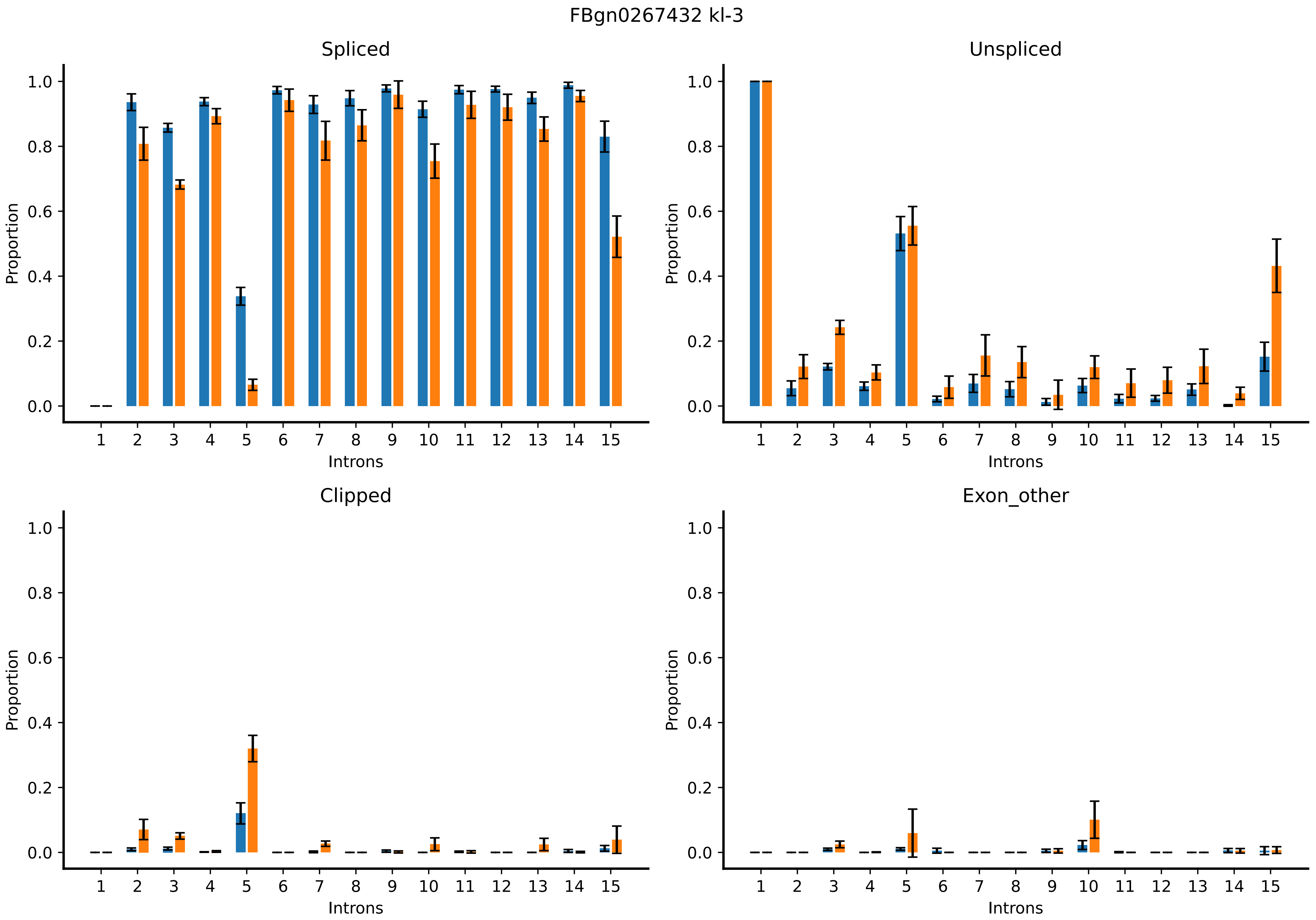
FBgn0267432 kl-3
Spliced
Unspliced
Clipped
Exon_other
Proportion
Introns
0.0
0.2
0.4
0.6
0.8
1.0
1
2
3
4
5
6
7
8
9
10
11
12
13
14
15

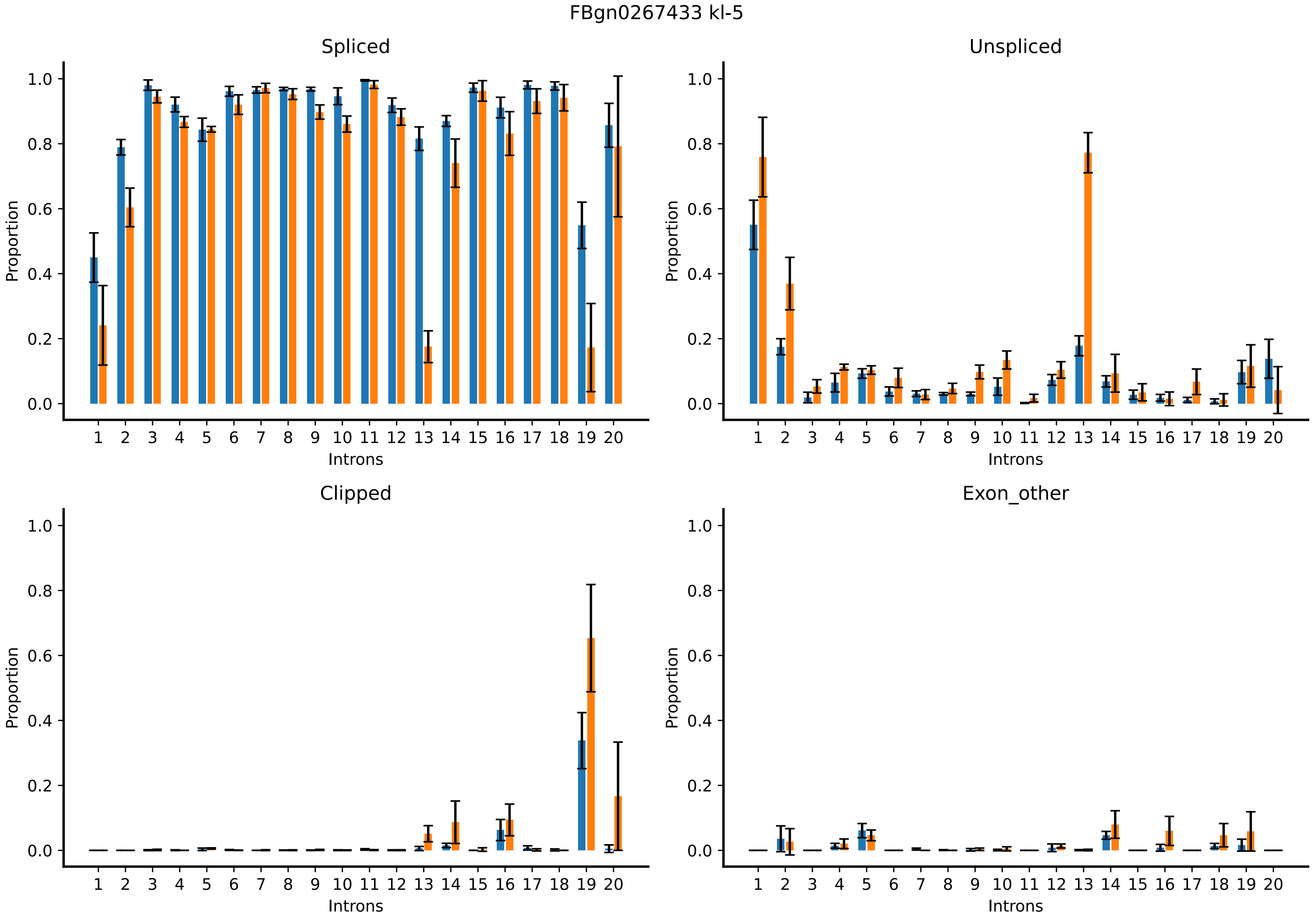

FBgn0267433 kl-5
Spliced
Unspliced
Clipped
Exon_other
Proportion
Introns
0.0
0.2
0.4
0.6
0.8
1.0
1 2 3 4 5 6 7 8 9 10 11 12 13 14 15 16 17 18 19 20

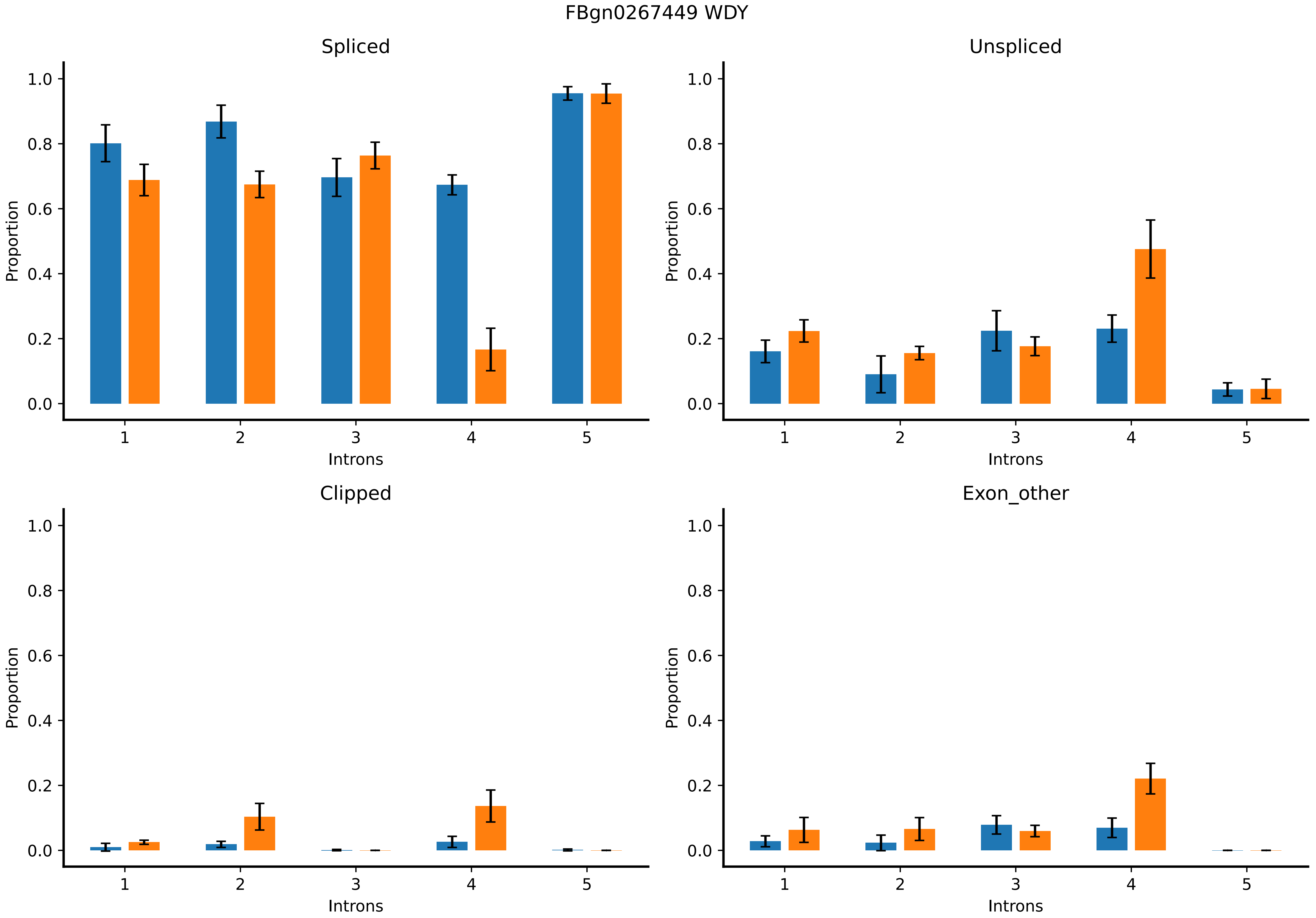
FBgn0267449 WDY
Spliced
Unspliced
Clipped
Exon_other
Proportion
Introns
0.0
0.2
0.4
0.6
0.8
1.0
1
2
3
4
5

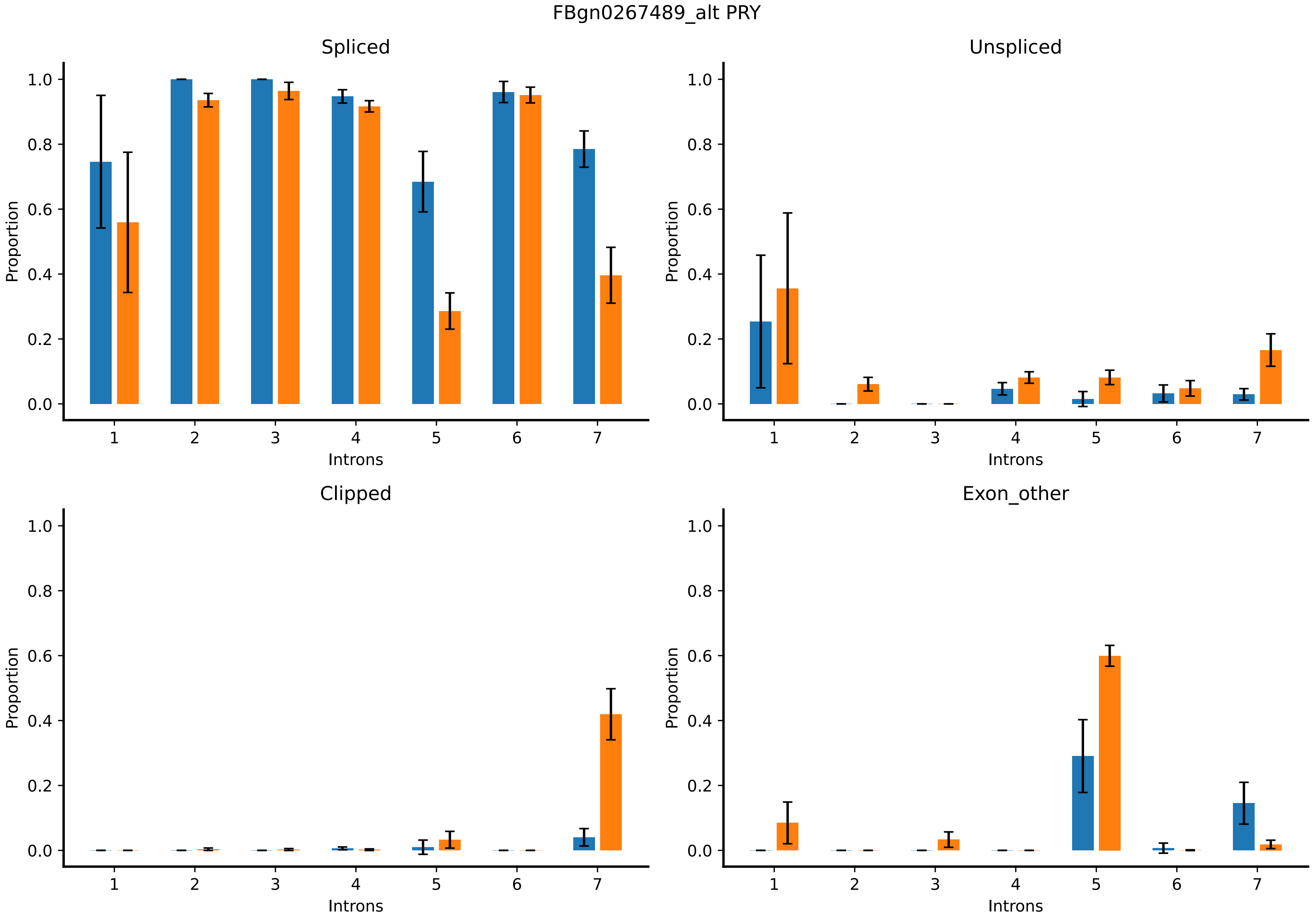
FBgn0267489_alt PRY
Spliced
Unspliced
Clipped
Exon_other
Proportion
Introns
0.0
0.2
0.4
0.6
0.8
1.0
1
2
3
4
5
6
7

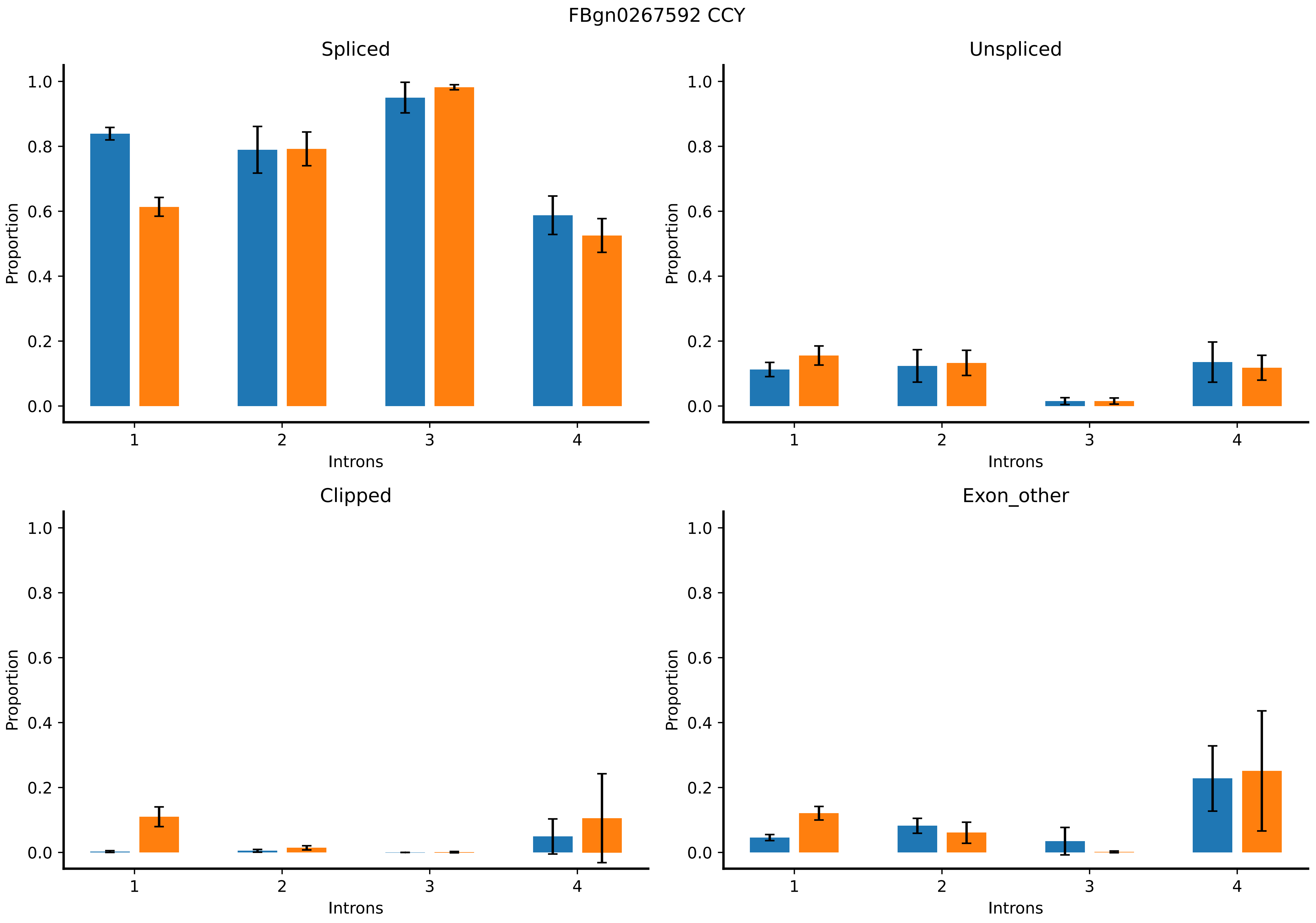
FBgn0267592 CCY
Spliced
Unspliced
Clipped
Exon_other
Proportion
Introns
0.0
0.2
0.4
0.6
0.8
1.0
1
2
3
4
